# Systematical analysis of ferroptosis regulators and identification of GCLM as a tumor promotor and immunological biomarker in bladder cancer

**DOI:** 10.3389/fonc.2022.1040892

**Published:** 2022-10-24

**Authors:** Song Wang, He Wang, Shaoxing Zhu, Fangyin Li

**Affiliations:** ^1^ Department of Urology, Cancer Hospital of University of Chinese Academy of Sciences (Zhejiang Cancer Hospital), Institute of Basic Medicine and Cancer (IBMC), Chinese Academy of Sciences, Hangzhou, Zhejiang, China; ^2^ The Second Clinical Medical College, Zhejiang Chinese Medical University, Hangzhou, Zhejiang, China

**Keywords:** ferroptosis, bladder cancer, immune infiltration, GCLM, biomarker

## Abstract

Bladder cancer (BCa) is a life-threaten disease with an increasing incidence with age, and immunotherapy has become an important treatment for BCa, while the efficiency of the immune system declines with age. It is vital to reveal the mechanisms of tumor immune microenvironment (TIME) and identify novel immunotherapy targets for BCa. Through analyzing the RNA-seq of TCGA-BLCA cohort, we distinguished two ferroptosis-related BCa clusters, and we discovered that in comparation with cluster 2, the cluster 1 BCa patients showed higher PD-L1 expression, more unfavorable overall survival and higher tumor stage and grade. XCELL analyses showed that higher level of Th2 cell and Myeloid dendritic cell were enriched in cluster 1, while NK T cell was enriched in cluster 2, and TIDE analysis revealed that cluster 2 was more sensitive to immunotherapy than cluster 1. GSEA analysis implied that Toll-like signaling pathway and JAK_STAT signaling pathway were significantly enriched in cluster 1. Subsequently, through performing bioinformatic analysis and cell experiments, we demonstrated that GCLM is overexpressed in BCa and indicates dismal prognosis, and knockdown of GCLM can significantly suppress the colony formation ability of BCa cells. Furthermore, we also found that GCLM might be correlated with immune infiltration in BCa, and can serve as a tumor promotor and immunological biomarker in BCa, our research showed the vital roles of ferroptosis regulators in TIME of BCa, and GCLM is a latent therapeutic target for cancer immunotherapy.

## Introduction

Bladder cancer is one of the most common malignancies, resulting in more than 550,000 new cases and 200,000 deaths annually worldwide (1). Despite remarkable advancements have been achieved in cancer treatment in recent years, the clinical outcomes of bladder cancer patients are still unsatisfactory, especially for the elderly (2). Recently, immunotherapies targeting immune checkpoints have shown unprecedented effects in treating multiple cancer, including the BCa (3). However, tumor immunosuppressive environment can greatly impair therapeutic effects and result in unsustainable clinical benefits (4). More and more studies demonstrated that combined therapies such as chemotherapy, radiotherapy and other targeted drugs could improve the tumor immune microenvironment (TIME) and enhance the therapeutic effects of immune‐checkpoint inhibitors (ICIs) (5, 6). Therefore, to probe the heterogeneity and potential mechanisms of TIME for BCa might help for personalized immunotherapy management.

Ferroptosis is a modifiable and complex programmed cell death process that requires accumulation of lipid peroxides (7). Despite the physio-pathologic roles of ferroptosis have not been known thoroughly, the mechanisms underlying ferroptosis have been revealed gradually with the help of great efforts from researchers. Given that the vital roles of ferroptosis in inducing cell deaths, ferroptosis might also play a key role in tumor progression (8). As a matter of fact, there are several studies have reported that the ferroptosis and its regulator genes could determine the destiny of bladder cancer cells (9, 10). However, most studies pay close attention to the intrinsic mechanisms of ferroptosis in BCa, the associations between ferroptosis and TIME still remain unclear. Emerging evidence indicates that the anti-PD-L1 antibodies could activate NK T cell thus induce cancer cells ferroptosis, while the ferroptosis depressor could restrain this process. Additionally, they also discovered a synergistic effect between ferroptosis activator and ICIs (anti-PD-L1 antibodies), they can synergistically inhibit cancer cell proliferation (11). However, a systematic analysis of ferroptosis regulators and TIME in BCa is still missing.

Recently, Liang et al. has constructed a ferroptosis-related genes (FRGs) signature to help predict the prognosis of BCa patients, while they did not cluster bladder cancer patients according to the expression of ferroptosis regulators (12). Here, we systematically analyzed the relative expression, prognostic values and cluster of ferroptosis regulator genes in BCa, we constructed two heterogenous BCa clusters and discovered different clinical characteristics and prognosis between two clusters, Distinct tumor immune microenvironment (TIME), PD-L1 expression and sensitivity to immunotherapy were also found between this two clusters, which will help to stratify different risk BCa patients and guide further treatment. Subsequently, the GCLM was determined as the potential key regulator among these ferroptosis genes, through performing bioinformatic analysis and cell experiments, we demonstrated that GCLM is overexpressed in BCa and indicates dismal prognosis, and knockdown of GCLM can significantly suppress the colony formation and migration ability of BCa cells. Furthermore, we also found that GCLM might be correlated with immune infiltration in BCa, and can serve as a tumor promotor and immunological biomarker in BCa. Our research provides a novel insight and potential therapeutic target for BCa.

## Materials and methods

### Human cell lines and qRT-PCR analysis

The human normal bladder cell line SV-HUC-1 (short name: SV) and BCa cell line UM-UC-3 (short name: UC3), T24 and J82 were obtained from Shanghai Institute of Cell Biology (Shanghai, China). The RPMI 1640 (for T24, J82 cell) and MEM (for UC3, SV cell) medium with 10% fetal bovine serum (FBS) were used to culture cells under a humidified atmosphere of 5% CO2 at 37°C. The TRIzol reagent (Takara) was used to extract total RNA, and PrimeScript RT Reagent Kit (Takara) was utilized to reverse into cDNA. The ABI 7500 fast real-time PCR System (Applied Biosystems) and SYBR Premix Ex Taq (Takara) was used to detect relative expression of GCLM mRNA *via* RT-qPCR method. GAPDH was the normalization reference. All primers were listed in (**Supplementary Table 1**).

### Cell transfection, trans-well and colony formation assay

The BCa T24/UC3 cells were treated with 50nM si-GCLM or si-NC, the transfection procedure was performed as previously described (13), and the si-RNAs were listed in (**Supplementary Table 1**). The transfected cells were cultured for 48h, and were digested in biosafety cabinet and calculated in cell counter (JIMBIO-FIL), the cells were then mixed with the culture medium according to the standard of 1000 cells/well, then added into a 6-well plate, the 6-well plate were cultured for 10 days, and were firstly fixed with methanol (15-20 min, 1ml/well), then stained with crystal violet (15-20 min, 1ml/well). Finally, the 6-well plate was rinsed in a container containing tap water, then dried and calculated to detect relative colony formation efficacy. The transwell compartments (Millipore) was used to evaluate the migration capacity of T24/UC3 cells, the BCa cells (about 7 × 104 UC3 and 5 × 104 T24 transfection cells) in serum-free medium (300 μL) were transferred to the upper layer of the chambers and put in 24-well plate (800μL 10% FBS medium) and cultured for 24 h under 37°C. Then the compartments were disposed with the methanol and crystal violet (0.1%). The phase-contrast microscope (Olympus, 20×) was used to image.

### Data acquisition and analysis

The clinical information and RNA-seq of TCGA-BLCA cohort (408 BCa and 19 normal bladder tissues) were derived from the TCGA database (https://portal.gdc.cancer.gov/), the HPA database (https://www.proteinatlas.org/) was utilized to assess the relative protein expression of GCLM in BCa, to reduce error, the same antibody (antibody ID: CAB009568) representative IHC stain image was selected, to externally validate the prognosis of GCLM in BCa, the Kaplan-Meier plotter (http://kmplot.com/analysis/) database was online analyzed.

### Consensus cluster analysis and LASSO regression analysis

Based on the expression of ferroptosis regulators in BCa, the consensus cluster analysis was performed through “Consensus Cluster Plus” package (the maximum number of clusters is six, and 80% of the total sample drawn 100 times), the clinical characteristics of two BCa clusters and Venn plot were displayed by using the Hiplot database (https://hiplot-academic.com/). The hub regulator gene selection was performed with the help of least absolute shrinkage and selection operator (LASSO) regression algorithm (“glmnet” package), and 10-fold cross-validation was used.

### GSEA analysis

The “Cluster Profiler” package was applied to perform the GSEA analysis and probe the mechanisms of GCLM and two clusters, the KEGG analysis was used to illustrate potential signaling pathway led to the distinct biological process, FDR and adjust p value <0.05 indicate meaningful pathways.

### TIME analysis

With the help of “GSVA” package, the ssGSEA algorithm was used to evaluate the relations of GCLM and 3 co-expressed genes with tumor immune cells. The xCell algorithm of “immunedeconv” and “pheatmap” package was implemented to assess immune infiltrating degree of two clusters, xCell algorithm was also used to investigate the links of GCLM and TIME in more than 30 types of cancer. The Tumor Immune Dysfunction and Exclusion (TIDE) analysis was used to predict ICB response (14). The expression relations of GCLM and immune checkpoints were also performed to illustrate the correlations of GCLM with TIME.

### Statistical analysis

The GraphPad Prism (8.0) and R (4.0.3) were applied to perform statistical analyses. The associations of GCLM expression with co-expressed genes and PD-L1 expression were evaluated by using spearman correlation analysis, the t test and Kruskal-Wallis test were used to perform comparison among groups. p < 0.05 indicated significance.

## Results

### Diverse expression patterns of ferroptosis regulators in BCa

**Figure 1:**
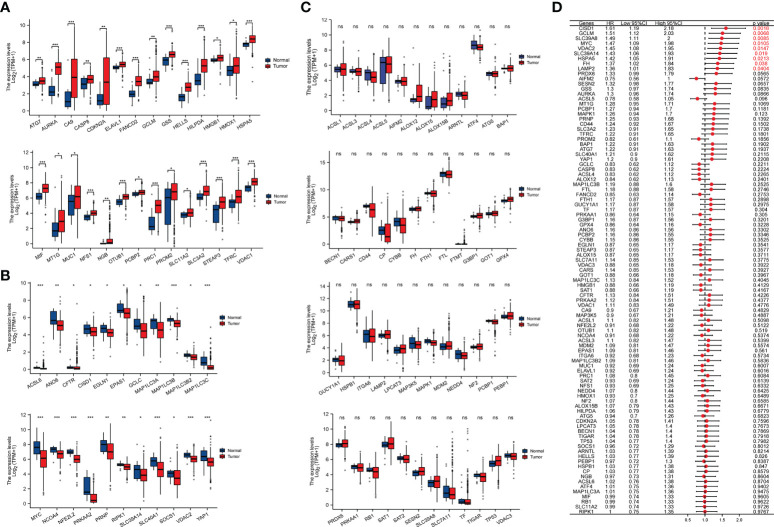
The relative expression levels and prognostic values of ferroptosis regulators in TCGA-BLCA cohort. **(A)** 28 significantly upregulated ferroptosis regulators in BCa. **(B)** 22 significantly downregulated ferroptosis regulators in BCa. **(C)** The expression levels of 48 ferroptosis regulators were not significantly different between BCa and normal bladder tissues. **(D)** The prognostic values of ferroptosis regulators in BCa, the red font represents statistical significance. *p < 0.05; **p < 0.01, ***p < 0.001. NS, Non Significance.

According to the previous studies, we selected 98 ferroptosis regulators to perform analysis, which were validated to play pivotal roles in cell ferroptosis (15, 16). Through analyzing the relative expression levels and prognostic values of ferroptosis regulators in TCGA-BLCA cohort, we found that there are 28 significantly upregulated ferroptosis regulators in BCa, including the ATG7, AURKA, CA9, CASP8, CDKN2A, ELAVL1, FANCD2, GCLM, GSS, HELLS, HILPDA, HMGB1, HMOX1, HSPA5, MIF, MT1G, MUC1, NFS1, NGB, OTUB1, PCBP2, PRC1, PROM2, SLC11A2, SLC3A2, STEAP3, TFRC and VDAC122 (P<0.05; **Figure 1A**), and there were 22 significantly downregulated ferroptosis regulators in BCa, including the ACSL6, ANO6, CFTR, CISD1, EGLN1, EPAS1, GCLC, MAP1LC3A, MAP1LC3B, MAP1LC3B2, MAP1LC 3C, MYC, NCOA4, NFE2L2, PRKAA2, PRNP, RIPK1, SLC39A14, SLC40A1, SOCS1, VDAC2, YAP1 (P<0.05; **Figure 1B**), and 48 non-differentially expressed ferroptosis regulators in BCa (P>0.05; **Figure 1C**). Furthermore, correlations of theses ferroptosis regulatory genes with the overall survival (OS) of BCa patients were also explored *via* using the Cox regression method, and the results implied that the highly expressed CISD1, GCLM, SLC39A8, MYC, VDAC1, SLC39A14, HSPA5, FH and LAMP2 were significantly associated with the worse OS of BCa patients (**Figure 1D**). These findings revealed that ferroptosis regulators may be involved in the progression of BCa.

### Identification of two heterogeneous clusters for BCa based on the expression levels of ferroptosis regulators

**Figure 2:**
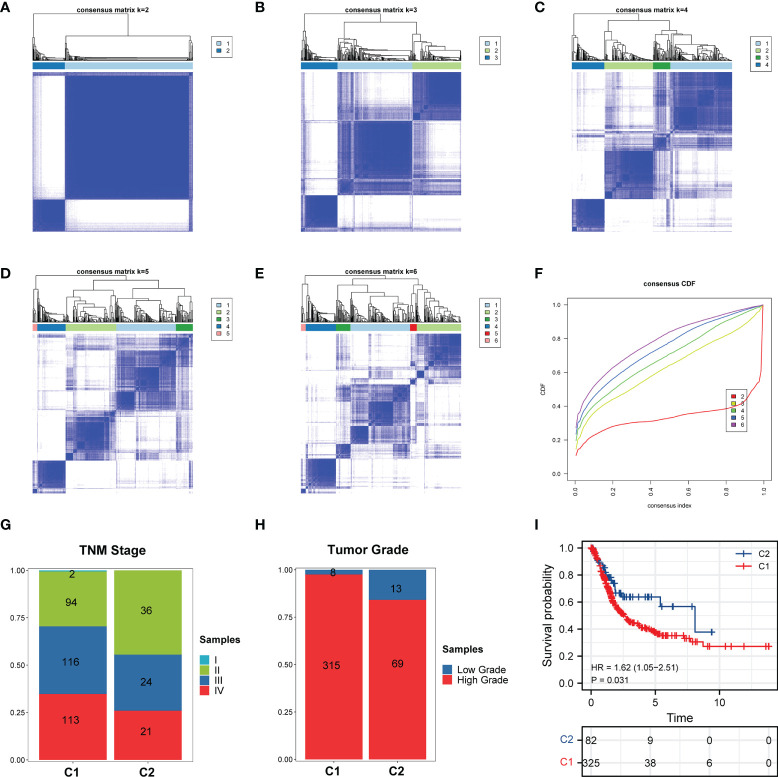
Consensus clustering analysis of ferroptosis regulators in BCa. **(A–E)** Consensus clustering matrix for **(A)** k = 2, **(B)** k = 3, **(C)** k = 4, **(D)** k = 5, **(E)** k = 6. **(F)** Cumulative curves for k = 2–6. **(G, H)** Distribution of C1/C2 in BCa **(G)** TNM stage and **(H)** tumor grade. **(I)** Kaplan-Meier curves showing OS of C1/C2. C1 and C2 represents Cluster 1 and Cluster 2, respectively.

Based on the RNA-seq data of TCGA-BLCA, two heterogeneous clusters of BCa were identified *via* conducting the consensus clustering analysis according to the expression of these ferroptosis regulators, and the results demonstrated that the k=2 (**Figure 2A**) was more optimal than k=3, 4, 5, 6 (**Figures 2B–E**) for BCa, and this was consistent with the results of cumulative curves (**Figure 2F**). Hence, these 408 BCa patients were divided into two independent clusters (C1/C2; cluster 1/2). Subsequently, through analyzing the clinical information of these two cluster BCa patients, we found that patients in cluster 1 show higher percent of tumor stage and grade than cluster 2 (**Figures 2G, H**). Moreover, in comparation with the cluster 2 patients, cluster 1 patients possessed more unfavorable overall survival (**Figure 2I**).

### Distinct immune environment and PD-L1 expression in two BCa clusters

**Figure 3:**
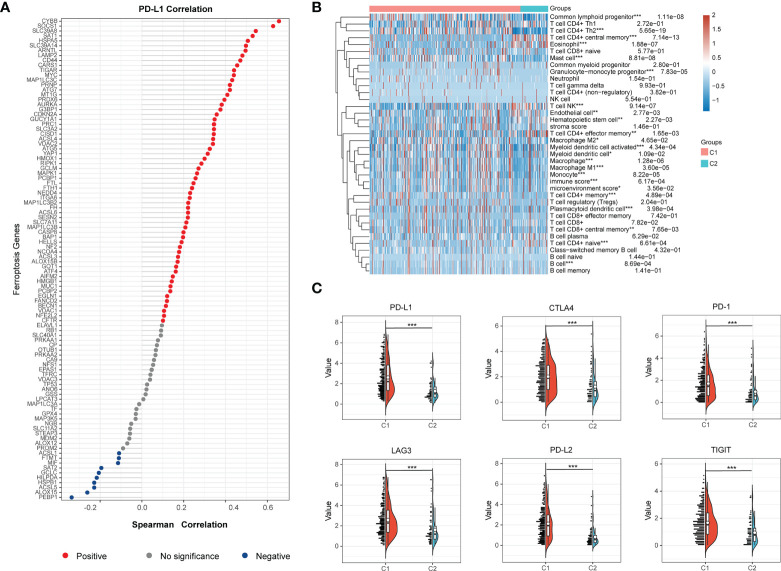
Distinct tumor immune microenvironment (TIME) between two BCa clusters. **(A)** Correlations of ferroptosis regulators with PD-L1 expression in BCa. The red dots represent positive correlation, grey dots represent no significance, and blue dots represent negative correlation. **(B)** The heatmap visualized distinct infiltrating level of immune cells between two BCa clusters. **(C)** The half-violin plots visualized the distribution of the immune checkpoints of two BCa clusters. *p < 0.05; **p < 0.01, ***p < 0.001.

**Figure 4:**
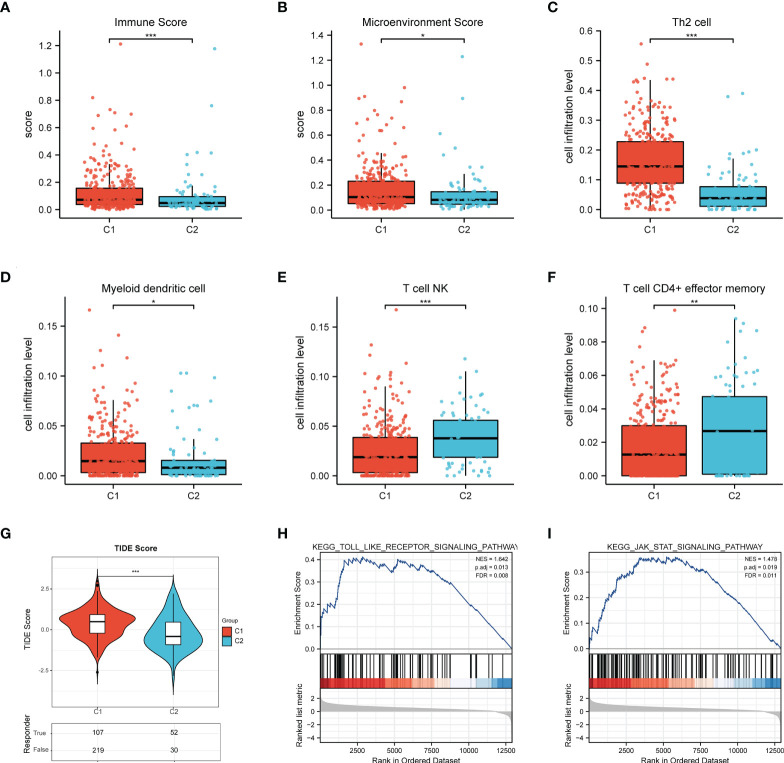
Differential infiltrating levels of immune cells between two BCa clusters. **(A)** The immune score and **(B)** microenvironment score of two BCa clusters. **(C-F)** The infiltrating levels of **(C)** Th2 cell, **(D)** Myeloid dendritic cell, **(E)** NK T cell, **(F)** CD4+ effector memory T cell in two clusters. **(G)** The TIDE score of C1/C2. **(H)** Toll-like signaling pathway and **(I)** JAK_STAT signaling pathway. *p < 0.05; **p < 0.01, ***p < 0.001.

In order to probe the immune microenvironment of the above two BCa clusters, we preliminarily analyzed the TCGA-BLCA cohort, and the results revealed that 62 ferroptosis regulators are positively correlated with PD-L1, 10 ferroptosis regulators are negatively correlated with PD-L1, and 26 ferroptosis regulators have no significant associations with PD-L1 (**Figure 3A**), this indicated that ferroptosis regulators may be tightly linked with the tumor immune microenvironment (TIME) of BCa. Therefore, we further explored the TIME of two clusters, and found that there is significantly distinct TIME between two BCa clusters (**Figure 3B**). We also investigate the expression of immune checkpoints of two clusters, and the results showed that the checkpoints such as PD-L1, CTLA4, PD-1, LAG3, PD-L2 and TIGIT were remarkably overexpressed in cluster 1 compared to cluster 2 (**Figure 3C**). Moreover, the results of xCell algorithm showed that the immune score and microenvironment score in cluster 1 are higher than cluster 2 (**Figures 4A, B**), for tumor-related immune cells, the Th2 cell and Myeloid dendritic cell were found to be remarkably enriched in cluster 1 (**Figures 4C, D**), while the NK T cell and CD4+ effector memory T cell were remarkably enriched in cluster 2 (**Figure 4E, F**). Additionally, we also evaluate the immune checkpoint blockade (ICB) response efficacy of two clusters *via* performing the TIDE analysis, and we found that the TIDE score of cluster 1 is significantly higher than cluster 2 (**Figure 4G**). Furthermore, the GSEA analysis was then applied to uncover the underlying mechanisms of ferroptosis regulators, and the results implied that the Toll-like signaling pathway and JAK_STAT signaling pathway were significantly enriched in cluster 1 (**Figures 4H, I**). These findings revealed that distinct TIME between two clusters.

### Identification of GCLM as the potential immune-related ferroptosis regulator in BCa.

**Figure 5:**
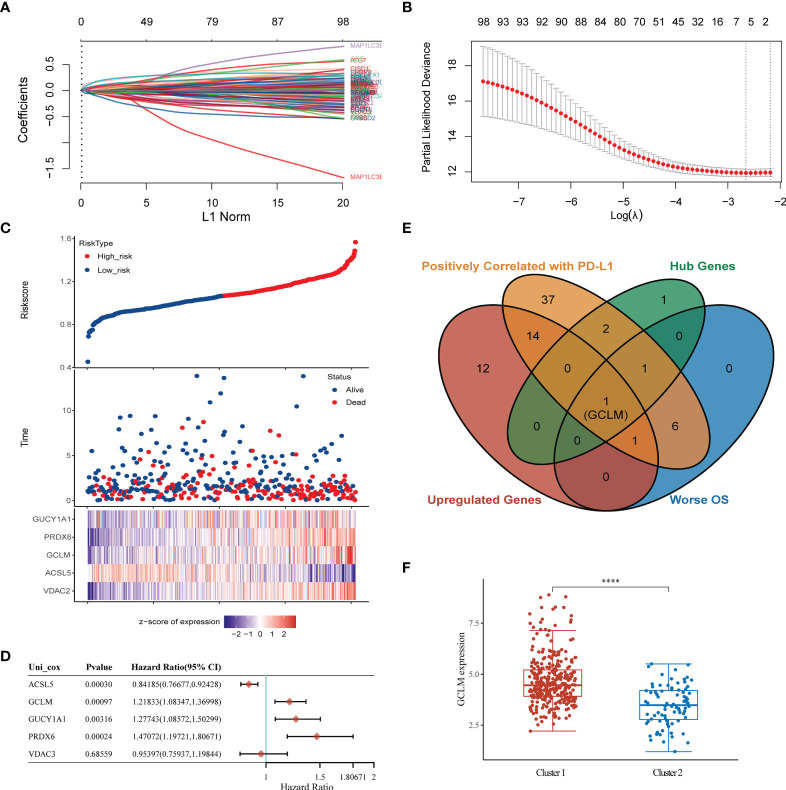
Identification of GCLM as the potential immune-related ferroptosis regulator in BCa. **(A)** Lasso regression of ferroptosis regulators in BCa. **(B)** The optimal lambda value in Lasso model. **(C)** Prognostic analysis of gene signature including 5 hub ferroptosis regulators. **(D)** The prognosis of 5 hub genes in BCa *via* Cox regression method. **(E)** A Venn diagram displayed that GCLM is the potential key ferroptosis regulator in BCa. **(F)** The relative expression of GCLM in Cluster 1 and 2. ****p < 0.0001.

**Figure 6:**
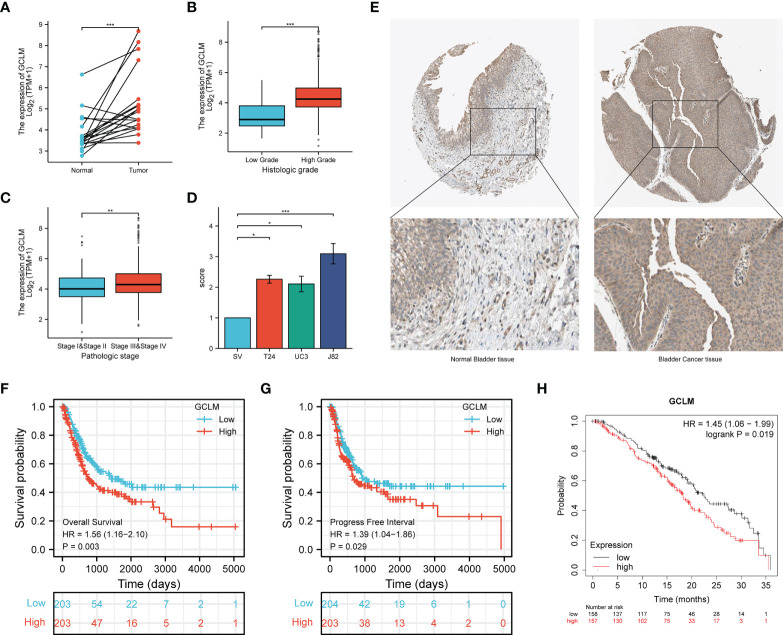
GCLM is highly expressed in BCa and its elevated expression correlated with unfavorable prognosis. **(A)** GCLM is highly expressed in 19 paired BCa and normal bladder tissues in TCGA-BLCA cohort. GCLM expression is associated with higher tumor histologic grade **(B)** and pathologic stage **(C)** in BCa. **(D)** GCLM is highly expressed in BCa T24, UC3 and J82 cell lines compared to normal bladder cell line SV. **(E)** The IHC stain of GCLM in normal bladder tissue (left) and bladder cancer tissue (right). Overexpression of GCLM predicts unfavorable **(F)** OS and **(G)** PFI in TCGA-BLCA cohort. **(H)** Overexpression of GCLM predicts unfavorable OS of BCa patients in Kaplan-Meier plotter database. *p < 0.05; **p < 0.01, ***p < 0.001.

To determine the key ferroptosis regulators of BCa, we conducted the LASSO regression analysis (**Figure 5A**), and found that the optimal lambda value in Lasso model is 5 (**Figure 5B**), which indicated that 5 ferroptosis regulators were the key factors among these regulators, including the GUCY1A1, PRDX6, GCLM, ACSL5 and VDAC3. Based on the expression of these 5 key regulators in BCa, we then constructed a prognostic signature, namely high and low risk-score group, which will help to make clinical decision to predict the prognosis for BCa patients (**Figure 5C**). Moreover, among these 5 key regulators, the overexpression of GUCY1A1, PRDX6, GCLM and reduction of ACSL5 could predict unfavorable OS for BCa patients (**Figure 5D**). Subsequently, to identify the potential immune-related hub ferroptosis regulator in BCa, we took an intersection of ferroptosis regulators that are positively correlated with the expression of PD-L1, associated with worse OS and highly expressed key regulators in BCa, and the results revealed that the GCLM may be the potential immune-related ferroptosis regulator in BCa (**Figure 5E**). Moreover, compared with the cluster 2, GCLM was also remarkably upregulated in cluster 1 (**Figure 5F**). To ulteriorly explore the expression and prognostic values of GCLM in BCa, we firstly analyzed the TCGA-BLCA dataset, and found that GCLM was remarkably upregulated in BCa tissues compared to normal bladder tissues (**Figure 6A**), and the expression of GCLM was increased along with the higher tumor grade and stage (**Figures 6B, C**). We then verified the relative expression of GCLM in BCa cells and normal bladder cell, and found that in comparation with the normal bladder cell (SV), the GCLM is significantly highly expressed in T24, UC3 and J82 BCa cells (**Figure 6D**), and the IHC intensity of GCLM in BCa tissue tended to stronger than normal bladder tissue (**Figure 6E**). Furthermore, through analyzing the clinical data of TCGA-BLCA cohort, we found that overexpression of GCLM predicts unfavorable OS and PFI (progression free interval) for BCa patients (**Figures 6F, G**), meanwhile, we also found that the highly expressed GCLM predicts unfavorable OS of BCa patients in Kaplan-Meier plotter database (**Figure 6H**). These findings implied that GCLM may be an important regulator in BCa progression.

### GCLM promote tumor progression and is associated with immune infiltration

**Figure 7:**
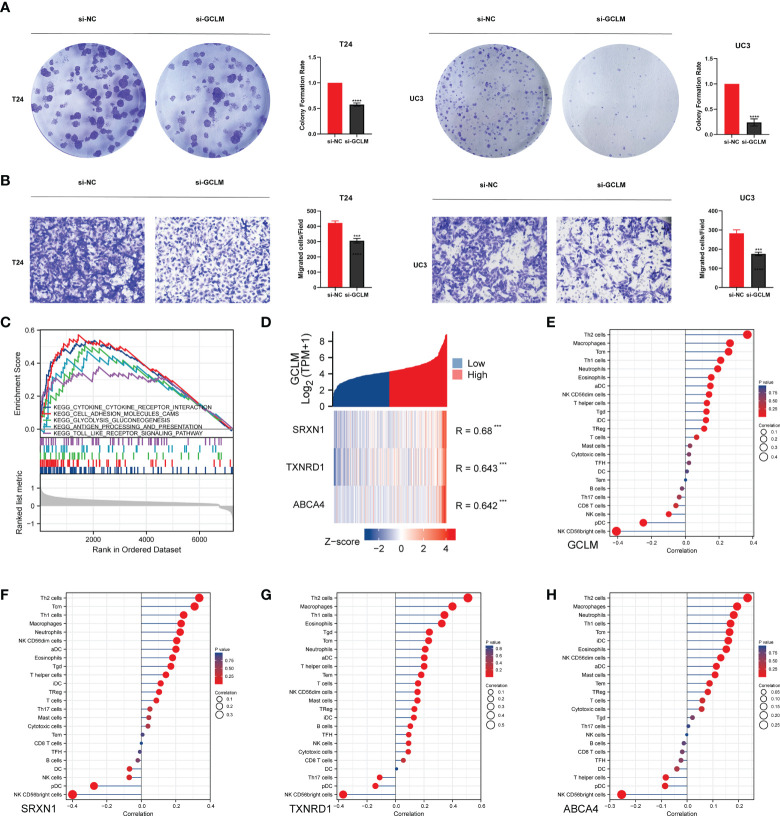
GCLM promotes tumor progression and is associated with immune infiltration in BCa. **(A, B)** Knockdown the expression of GCLM can significantly inhibit the **(A)** colony formation ability and **(B)** migration ability of T24 and UC3 cells. **(C)** The results of GSEA algorithm analysis indicated the potential KEGG signaling pathways of highly expressed GCLM in BCa. **(D)** The heatmap indicated that the SRXN1, TXNRD1 and ABCA4 are top 3 GCLM co-expressed genes in TCGA-BLCA cohort. The ssGSEA analysis of the correlations of **(E)** GCLM, **(F)** SRXN1, **(G)** TXNRD1 and **(H)** ABCA4 with immune cells in BCa. ***p < 0.001, ****p < 0.0001.

**Figure 8:**
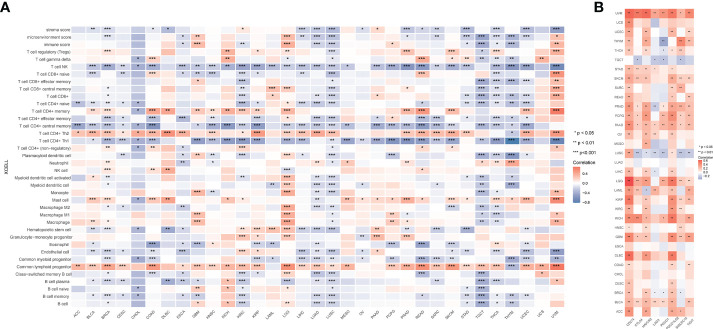
Systematic analysis of the links between GCLM expression and immune infiltration in pan-cancer. **(A)** XCELL analysis the links between GCLM expression and immune cells in pan-cancer. **(B)** Correlations of GCLM and immune checkpoints in pan-cancer. *p < 0.05; **p < 0.01, ***p < 0.001.

Given that GCLM is highly expressed in BCa, we then silenced the endogenous expression of GCLM in BCa T24/UC3 cells and performed the colony formation and trans-well assays, and the results showed that the colony formation and migration ability of si-GCLM treated T24 and UC3 cells were significantly inhibited compared to si-NC treated cells (**Figures 7A, B**). Subsequently, we applied GSEA analysis to explore the potential mechanisms of GCLM in BCa, and we found that the cytokine-cytokine receptor interaction pathway, cell adhesion molecules cams pathway, glycolysis gluconeogenesis, antigen processing and presentation, and toll like receptor signaling pathway were significantly enriched in highly expressed GCLM BCa samples (**Figure 7C**). Furthermore, we also performed the correlation analysis of GCLM in TCGA-BLCA cohort, and the results showed that SRXN1, TXNRD1 and ABCA4 are the top 3 GCLM co-expressed genes (**Figure 7D**). Moreover, we also found that GCLM and its 3 co-expressed genes were tightly linked with the infiltrating levels of immune cells, especially the Th2 cell and macrophages (**Figures 7E–H**). Additionally, we also systematically analyzed the links between GCLM expression and tumor immune infiltration in pan-cancer, and the results implied that high expression of GCLM tightly linked with the infiltrating level of Th2 cell in various types of cancer, while negatively correlated with the infiltrating levels of NK T cell in multiple cancers, including the BCa (**Figure 8A**). Meanwhile, we also explored the associations between 8 common immune checkpoints and GCLM in pan-cancer, and found that GCLM tightly linked with the expression of immune checkpoints in cancers, especially the CD274 (also namely PD-L1) (**Figure 8B**). Collectively, these findings revealed that overexpressed GCLM was tightly linked with immune infiltrating in cancers, including the BCa.

## Discussion

Ferroptosis has attracted much attention since 2012 for its associations with aging, immunity and cancer (7). Emerging evidence reports the crucial roles of ferroptosis in the development of human malignancies (17), while the potential roles of ferroptosis in regulating TIME of BCa are still missing. Here, we found that the ferroptosis regulators are frequently aberrant expressed in BCa, then we determined two heterogeneous clusters for BCa based on the expression levels of ferroptosis regulators, and found distinct prognosis and TIME between the two clusters, among these ferroptosis regulators, we further identified the GCLM as the key immune-related regulator, and knockdown its expression in BCa cells could significantly inhibit tumor progression.

Recently, the treatment for human cancers have gradually evolved with the proposition and development of precision medicine (18). Tumors can be defined as specific subtypes with different sensitivity to some therapeutic methods (e.g radiotherapy, immuno-therapy or chemotherapy) based on the big data analysis for patients, thus implementing individualized treatment (19). There are many studies attempted to distinguish specific patients that are susceptible to immunotherapy (e.g ICIs) (20, 21). For example, some researchers classified the Lung adenocarcinoma (LUAD) patients into two independent clusters with different clinical characteristics and clinical outcomes according to the expression of immune genes in LUAD, and their research showed that the high-risk immune LUAD patients could obtain more durable benefits from ICIs than low-risk patients (22). Meanwhile, the colorectal cancer patients were also divided into two independent subtypes based on the expression of autophagy genes, namely low and high-risk patients, and their study revealed that high-risk group patients are more susceptible to aggressive and targeted therapies (23). Recently, some studies have focused on the exploration of the effects of ferroptosis on the development of BCa. Liang et al. constructed a ferroptosis-related genes (FRGs) signature to help predict the prognosis of BCa patients, while they did not cluster bladder cancer patients according to the expression of ferroptosis regulators. they used the Cox regression analysis to construct a 7 ferroptosis-related genes (FRGs) risk score to help predict prognosis for BCa patients based on the expression of 60 ferroptosis genes. Interestingly, Despite the number of ferroptosis genes (we included 98 ferroptosis genes) included in the study varied, their study also identified the GCLM as one hub ferroptosis genes among these 7 FRGs, which also indicates the potentially important role of GCLM in the development of BCa (12). Xia et al. established a ferroptosis score to quantify the prognosis and therapeutic effects of BCa patients based on the expression of ferroptosis genes, they also two distinct BCa clusters based on expression of ferroptosis genes, while they did not identify the potential hub ferroptosis regulator among these genes (24). Here, according to the expression patterns of ferroptosis regulators in BCa, two heterogeneous clusters were identified, in comparation with cluster 2, the cluster 1 BCa patients showed higher PD-L1 expression, more unfavorable overall survival and higher tumor stage and grade, and TIDE analysis revealed that cluster 2 was more sensitive to immunotherapy than cluster 1. This classification scheme could help to make clinical decision for BCa treatment. Meanwhile, XCELL analyses showed that higher level of Th2 cell and Myeloid dendritic cell were enriched in cluster 1, while NK T cell was enriched in cluster 2, emerging evidence suggests that anti-PD-L1 antibodies with NK T cell could promote the ferroptosis process of cancer cells, while the inhibitor of ferroptosis could depress this combination effects in tumor cells (11, 25). These findings implied overwhelmingly complex functions in regulating TIME of BCa. Furthermore, the GSEA analysis was carried out to investigate the underlying mechanisms of this two clusters, the results implied that Toll-like signaling pathway and JAK_STAT signaling pathway were significantly enriched in cluster 1. Numerous studies reported that the Toll-like signaling pathway are important regulators of human immune system, its activation could evoke specific immune responses (26). Moreover, the abnormal activation of JAK_STAT signaling pathway was demonstrated to be participated in immunologic derangement and the occurrence of cancer (27). These findings implied that the interactions of ferroptosis with Toll-like signaling pathway and JAK_STAT signaling pathway might play vital roles in regulating TIME of BCa.

Subsequently, through performing cell experiments and bioinformatic analyses, GCLM was determined as the potential key regulator among these ferroptosis genes. GCLM is an important regulator for ferroptosis, it is also the firstly reported rate‐limiting enzyme of glutathione synthesis (28). Harris et al. demonstrated that the GCLM driven synthesis process of the antioxidant glutathione (GSH) is essential for tumor occurrence (29). Inoue et al. reported that the inhibition of GCLM could improve the CDDP-resistance process in non-small cell lung cancer, and it can serve as a potential target for treatment (30). However, the interactions of TIME and GCLM in BCa are still missing. Hence, we also attempted to probe the functions and mechanisms of GCLM in BCa, we demonstrated that GCLM is overexpressed in BCa and its overexpression indicates dismal prognosis, knockdown of GCLM can significantly suppress the colony formation ability and migration ability of BCa cells, we also found that GCLM might be correlated with immune infiltration in BCa, and can serve as a tumor promotor and immunological biomarker in BCa. Our research provides a novel insight and potential therapeutic target for BCa. However, there are also some defects in our study. Firstly, 98 regulators of ferroptosis were based on literature review, it is possible that some potential unknown regulators were not included in this study. Secondly, we conducted the clustering analysis and immune analyses were based on TCGA-BLCA cohort, because of lacking samples in our own center, more bigger cohort analyses are needed to verified out findings. Finally, although we performed the *in vitro* assay to illustrate the tumor promotor role of GCLM in BCa, more assayed are also needed to further demonstrate the role and mechanism of GCLM in BCa.

Collectively, our research comprehensively explored the expression patterns, diagnosis of ferroptosis regulators in BCa, and two clusters were constructed to help clinical decision making, we also found that the GCLM might be a tumor promotor and immunological biomarker in BCa.

## Data availability statement

The datasets presented in this study can be found in online repositories. The names of the repository/repositories and accession number(s) can be found in the article/**Supplementary Material**.

## Author contributions

SW and HW performed the experiments and bioinformatics analysis, SW wrote the manuscript. FL and SZ searched literatures, SW and FL revised the manuscript. All authors contributed to the article and approved the submitted version.

## Funding

This study was supported by Zhejiang Province Chinese medicine scientific research fund (2021ZA021).

## Conflict of interest

The authors declare that the research was conducted in the absence of any commercial or financial relationships that could be construed as a potential conflict of interest.

## Publisher’s note

All claims expressed in this article are solely those of the authors and do not necessarily represent those of their affiliated organizations, or those of the publisher, the editors and the reviewers. Any product that may be evaluated in this article, or claim that may be made by its manufacturer, is not guaranteed or endorsed by the publisher.
