# Supplementary material for: Systematical analysis of ferroptosis regulators and identification of GCLM as a tumor promotor and immunological biomarker in bladder cancer
Source: Front Oncol. 2022 Oct 24;12:1040892. doi: 10.3389/fonc.2022.1040892 (PMC9638099; doi:10.3389/fonc.2022.1040892)
Supplement: Supplementary file 1 [file Table_1.docx]

| **Table S1.** **The Oligonucleotides used in this study.** | | | |
| --- | --- | --- | --- |
| si-RNA | Sense (5'-3') |  |  |
| si-GCLM-1 | CCACCAGATTTGACTGCATTT | |  |
| si-GCLM-2 | CCAAATAGTAACCAAGTTAAT | |  |
| Primers | Forward Primer (5'-3') | | Reverse Primer (5'-3') |
| GCLM | TCAACCCAGATTTGGTCAGGGAGT | | TCCAGCTGTGCAACTCCAAGGA |
| GAPDH | CTGGGCTACACTGAGCACC | | AAGTGGTCGTTGAGGGCAATG |
